# A Seven-Gene Signature to Predict Prognosis of Patients With Hepatocellular Carcinoma

**DOI:** 10.3389/fgene.2021.728476

**Published:** 2021-09-16

**Authors:** Junli Wang, Qi Zhang, Fukang Shi, Dipesh Kumar Yadav, Zhengtao Hong, Jianing Wang, Tingbo Liang, Xueli Bai

**Affiliations:** ^1^Department of Hepatobiliary and Pancreatic Surgery, The First Affiliated Hospital, Zhejiang University School of Medicine, Hangzhou, China; ^2^Zhejiang Provincial Key Laboratory of Pancreatic Disease, The First Affiliated Hospital, Zhejiang University School of Medicine, Hangzhou, China; ^3^Cancer Center, Zhejiang University, Hangzhou, China; ^4^Clinical Research Center of Hepatobiliary and Pancreatic Diseases, Hangzhou, China

**Keywords:** Hepatocellular carcinoma, prognosis, differential expression genes, overall survival, model

## Abstract

**Purpose:** Hepatocellular carcinoma (HCC) is one of the most prevalent malignant diseases worldwide and has a poor prognosis. Gene-based prognostic models have been reported to predict the overall survival of patients with HCC. Unfortunately, most of the genes used in earlier prognostic models lack prospective validation and, thus, cannot be used in clinical practice.

**Methods:** Candidate genes were selected from GEPIA (Gene Expression Profiling Interactive Analysis), and their associations with patients’ survival were confirmed by RT-PCR using cDNA tissue microarrays established from patients with HCC after radical resection. A multivariate Cox proportion model was used to calculate the coefficient of corresponding gene. The expression of seven genes of interest (*MKI67, AR, PLG, DNASE1L3, PTTG1, PPP1R1A*, and *TTR*) with two reference genes was defined to calculate a risk score which determined groups of different risks.

**Results:** Our risk scoring efficiently classified patients (*n* = 129) with HCC into a low-, intermediate-, and high-risk group. The three groups showed meaningful distinction of 3-year overall survival rate, i.e., 88.9, 74.5, and 20.6% for the low-, intermediate-, and high-risk group, respectively. The prognostic prediction model of risk scores was subsequently verified using an independent prospective cohort (*n* = 77) and showed high accuracy.

**Conclusion:** Our seven-gene signature model performed excellent long-term prediction power and provided crucially guiding therapy for patients who are not a candidate for surgery.

## Introduction

Hepatocellular carcinoma (HCC) remains the fifth leading cause of cancer-related mortality worldwide (Sung et al., 2021). Although salient development has been achieved in the treatment of HCC, the 5-year overall survival rate is merely 18%. Surgical resection typically remains the most effective curative treatment, yet most of the patients inevitably suffer from the local recurrence (Llovet et al., 2004, 2016; Reau, 2020). HCC invariably presents significant inter-patient heterogeneity, and whether a certain patient with HCC can benefit from surgical resection or postoperative adjuvant therapies is ill-defined. This specific information has been important since it guides the clinical decision-making in the choice of the treatment. Conventionally, prediction of the prognosis of the patients with HCC was primarily relying on clinicopathologic parameters such as tumor size, lymph node, distant metastasis status, and pathological grade (Hao et al., 2009; Chan et al., 2018). Nevertheless, these prognostic factors cannot prop up the specificity and sensitivity of a meaningfully appreciable clinical model for HCC.

With the advent of the molecular signatures and high-throughput data platforms, several novel methods have been proposed as prognostic prediction models for the patients with HCC (Dvorchik et al., 2007; Marquardt et al., 2012). At present, the pretreatment neutrophil–lymphocyte ratio (Najjar et al., 2018; Hong et al., 2020), circulating tumor cells (Qi et al., 2018; Wang et al., 2020), and cancer-related differentially expressed genes (Sarathi and Palaniappan, 2019; Wang et al., 2019) have been used to predict the prognosis of patients with HCC, whereas most of those prediction models incorporated only one prognostic factor of HCC or lacking another prospective validation, resulting far away from the consensus of molecular prognostic prediction. Consequently, an objective and clinically ready prognostic prediction model is urgently required for the patients with HCC undergoing surgical resection, to determine whether or not adjuvant therapies are warranted for an individual patient.

Here, we developed a novel seven-gene expression signature to predict the overall survival of the patients with HCC by 129-paired tumor and normal cDNA tissue microarrays derived from frozen HCC samples. We carefully selected seven cancer-related differentially expressed genes varied from previously reported HCC prognostic models, which outstandingly performed to predict the overall survival. Surprisingly, they are associated with the tumor differentiation stage of patients with HCC. Furthermore, an independent prospective cohort including another 77 patients with HCC was used to validate the performance of this model.

## Materials and Methods

### Patients

For the construction of the model, frozen tissue of 378 paired tumor and normal samples were collected from the Second Affiliated Hospital, Zhejiang University School of Medicine between 2012 and 2018, who were all diagnosed with primary HCC by pathology and underwent curative surgical resection. The validation cohort containing 101 paired samples from patients with HCC was collected from the First Affiliated Hospital, Zhejiang University School of Medicine in 2019. All patients received follow-up every month for the first year and every 3–6 months thereafter. Survival time was calculated from the date of surgery to the date when death was confirmed. Patients with loss of follow-up or died of causes other than cancer were censored. This study was performed in accordance with the International Ethical Guidelines for Biomedical Research Involving Human Subjects and the principles expressed in the Declaration of Helsinki, and was approved by the ethic committee of the First and the Second Affiliated Hospital, Zhejiang University School of Medicine. Written informed consent was acquired from the patients and the patients’ parties. The details of involved patients’ clinical information are described in Supplementary Table 1.

### cDNA Tissue Microarray

In brief, total RNA was extracted from frozen tissue samples (about 1 cmł) using TriZol Reagent (Invitrogen) and the absence of DNA contamination was verified. Reverse transcription was performed by Prime Script^TM^ RT Reagent Kit (Takara Biotechnology Co., Dalian, China) following the manufacturer’s protocol. Finally, a cDNA tissue microarray with 186 paired samples was established, and samples were loaded in triplicate using 384-well plates.

### Development of the Seven-Gene Prognostic Expression Signature

Candidate genes were selected from Gene Expression Profiling Interactive Analysis (GEPIA) (Tang et al., 2017) with the following inclusion criteria: (1) the gene expression was significantly changed between tumor and normal tissue in HCC; (2) the gene expression was substantially associated with overall survival (OS) and progression-free survival (PFS) of HCC, even when compared with reference genes; (3) the quartile hazard ratio (HR) was higher than median HR and they were consistently varied; and (4) the expression of Transcripts Per Million (TPM) was greater than 10. Primers are presented in Supplementary Table 2. The expression of each gene was measured by quantitative reverse transcription-polymerase chain reaction in-house 186-paired cDNA tissue microarrays using TB green premix EX Taq^TM^ Reagent Kit (Takara Biotechnology Co., Dalian, China) following the manufacturer’s protocol, performed with Prism 7900HT instruments (Applied Biosystems).

Then, the gene expression value was normalized relative to a set of two reference genes *ACTB* and *GAPDH* and transformed into a log-2 base for further analysis. A univariate Cox hazard model was performed to identify the survival-associated differentially expressed genes satisfying the criteria of the hazard ratio (HR) > 1 or HR < 1 and a *p* value < 0.05. Subsequently, a multivariate Cox proportion model was used to calculate corresponding gene coefficients (Iasonos et al., 2008; Tibshirani, 1997). Ultimately, a multiple-gene signature for prognostic prediction in the training cohort was constructed where the risk score could be calculated using the following formula:


$$RiskScore\left(RS\right)=\sum_{n}^{i}\left(Expn\right)\left(\beta n\right)$$

Where β*n* is the regression coefficient of the *n*th gene and *Expn* is the log2-transformed expression value of the *n*th prognostic gene where β*N* is the regression coefficient of the Nth gene and ExN is the log2-transformed expression value of the *n*th prognostic gene.

### Statistical Analyses

Data analysis was performed using Prism 8 software (GraphPad, San Diego, CA, United States). Survival data were presented with Kaplan–Meier curves and analyzed using a log-rank test. The multivariate Cox proportion model was performed by STATA software (version 13) to define risk categories. For all tests, a *p*-value less than 0.05 was considered statistically significant.

## Results

### Identification of Potential Genes

**FIGURE 1:**
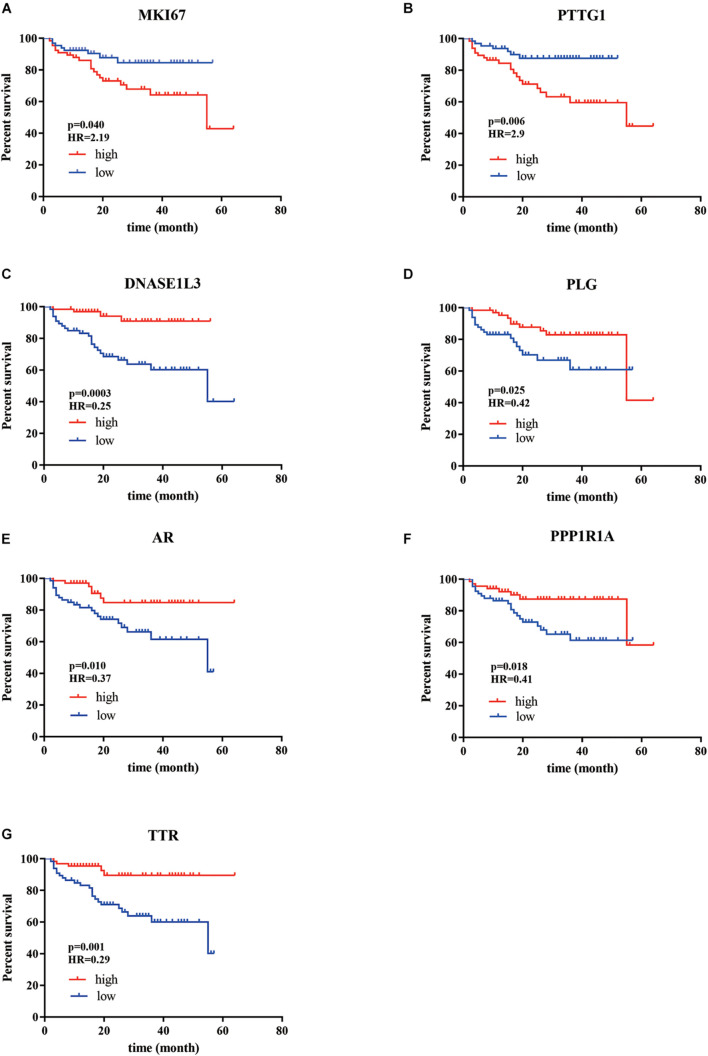
The Kaplan–Meier analysis identified relevant prognostic genes from patients with HCC after radical resection. **(A)** *MKI67*; **(B)** *PTTG1*; **(C)** *DNASE1L3*; **(D)** *PLG*; **(E)** *AR*; **(F)** *PPP1R1A*; **(G)** *TTR*. The red lines indicate cases with high expression (expression value > median), while the blue line indicates cases with low expression (expression value < median). HR, hazard ratio; *MKI67* (a marker of proliferation Ki-67), *PTTG1* (*PTTG1* regulator of sister chromatid separation, securin), *DNASE1L3* (deoxyribonuclease 1 like 3), *PLG* (plasminogen), *AR* (androgen receptor), *PPP1R1A* (protein phosphatase 1 regulatory inhibitor subunit 1A), and *TTR* (transthyretin). *P*-values were obtained from the log-rank test.

According to our criteria, a total of 15 genes involving cell proliferation, invasion, hormone, and those with significantly differential expression between tumor and normal tissue were selected. We then tested the correlation between the expression of these selected genes and patients’ survival using our cohort. It turned out that the expression of seven genes was found to be significantly associated with OS (Figure 1), including marker of proliferation Ki-67*-MKI67* (HR = 2.19, *p* = 0.04), androgen receptor-*AR* (HR = 0.37, *p* = 0.01), plasminogen-*PLG* (HR = 0.42, *p* = 0.0025), deoxyribonuclease 1 like 3-*DNASE1L3* (HR = 0.25, *p* = 0.0003), *PTTG1* regulator of sister chromatid separation, securin-*PTTG1* (HR = 2.9, *p* = 0.006), protein phosphatase 1 regulatory inhibitor subunit 1A-*PPP1R1A* (HR = 0.41, *p* = 0.018), and transthyretin-*TTR*. Among the seven genes, MKI7 and PTTG1 were negatively correlated with OS, and the others were significantly associated with longer survival of the patients with HCC.

### Survival Prognostic Prediction Model Construction

The above HRs were subsequently used to construct a comprehensive model by multiple regression (proportional hazards regression), in which the contribution of an individual gene was calculated as HR coefficients. Consequently, a risk score for prognosis prediction was calculated as follows: Risk score = (0.368 × ExpMKI67 + 1.082) + (−0.719 × ExpPTTG1 + 0.424) + (0.286 × ExpDNASE1L3 -1.276) + (0.204 × ExpPLG + 0.172) + (0.173 × ExpAR - 0.702) + (0.153 × ExpPPP1R1A - 0.731) + (0.286 × ExpTTR - 0.779), where Exp stands for the expression level of each gene that was normalized to reference genes. We endowed each patient with a specific risk score, which ranged between 1 and 14 (Table 1).

**TABLE 1:** Overall survival score risk categories of primary HCC patients after curative surgical resection.

Risk category	Numbers of patients	Percentage of patients (%)	Overall survival score (range 1–14)
Low	15	12	<5
Intermediate	78	60	5–9
High	12	28	>9

**FIGURE 2:**
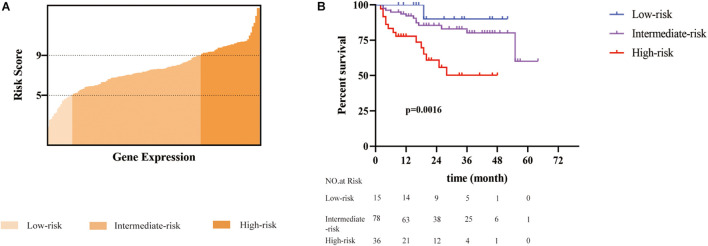
A seven-gene signature defined risk scoring was constructed to predict the overall survival of patients with HCC after radical resection. **(A)** A total of 129 patients was divided into three groups based on the risk score (ranged from 1 to 14). Patients with a risk score of less than 5 were defined as low risk. Patients with a risk score between 5 and 9 were classified into the intermediate-risk group. Patients with a risk score greater than 9 were defined as a high risk. **(B)** The difference in overall survival among the three groups was statistically significant (*p* = 0.0016) performed by multivariate Cox proportion model.

In the training cohort, patients were assigned into three groups according to their risk score (Figure 2A): low risk (with a risk score less than 5, *n* = 15, accounting for 12%), intermediate risk (with a risk score between 5 and 9, *n* = 78, accounting for 60% of the whole cohort), and high risk (with a risk score higher than 9, *n* = 36, accounting for 28%). The cutoff values were finally chosen dependent on the optimal patient proportion and significant separation of survival curve after we tried different segmentation points. The Cox model based on the seven-gene expression signature supplied substantial prediction power (Figure 2B). Accordingly, the 1-year OS rates in the low-, intermediate-, and high-risk group, respectively, were 100, 90.5, and 61.9%. Furthermore, the three groups showed more meaningful distinction of 3-year OS rates, which were 88.9 and 74.5% in low- and intermediate-risk groups, and only 20.6% in the high-risk group. The 5-year OS rates presented similar differences, which were 88.9, 71.2, and 20.6% separately in the low-, intermediate-, and high-risk group. Median OS for the high-risk group was 16 months versus 27 and 21 months in the low- and intermediate-risk group. Although disease-free survival among these groups was comparable, the 5-year recurrence rates of the low-, intermediate-, and high-risk group were 26.7, 25.6, and 33.3%, respectively (Supplementary Figure 1A).

### Validation of the Seven-Gene Signature Model

**FIGURE 3:**
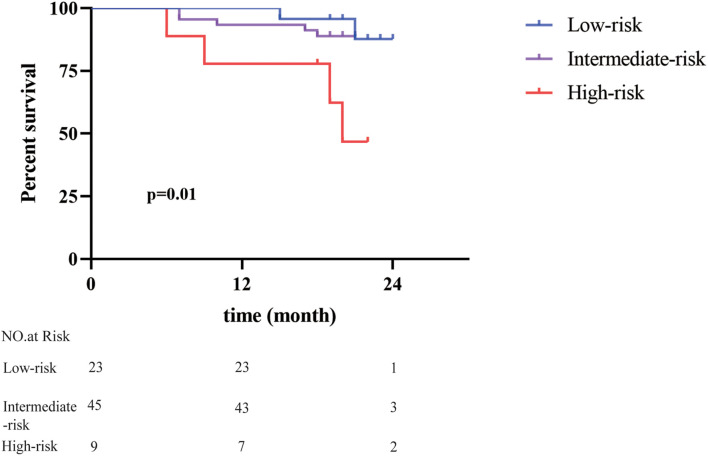
Validation of predictive value of the seven-gene signature. The risk scores of 77 patients with HCC in an independent cohort were calculated. Patients with different risk scores had distinct overall survival after radical resection (*p* = 0.01).

To verify the power of our model, we used a separate cohort containing 77 patients with HCC for external validation. These patients were also classified into the low-risk group (*n* = 23, accounting for 30%), intermediate-risk group (*n* = 45, accounting for 58%), and high-risk group (*n* = 9, accounting for 12%) based on the calculated prognostic score. Survival analysis indicated that the patients in the high-risk group showed a much poorer prognosis than the other two groups (Figure 3). The 1-year OS rates for the low-, intermediate-, and high-risk group were 100, 93.0, and 71.4%, respectively. Furthermore, the high-risk group presented evident differences in 2-year OS rates of 42.9% compared with 90.5 and 88.2% in the low- and intermediate-risk group. Meanwhile, the cohort was categorized into a low-, intermediate-, and high-risk group to predict the 3-year recurrence rate, which were 13.0, 22.2, and 33.3%, respectively (Supplementary Figure 1B).

We further investigated the correlation between OS and traditional clinicopathological parameters in the current two cohorts. Tumor size was not well associated with OS (Supplementary Figures 2A,B). Tumor differentiation showed significant differences in OS among the three groups in one cohort but not in the other, suggesting that it was not robust as a predictor of prognosis in patients with HCC (Supplementary Figures 2C,D). Meaningfully, our risk score showed a positive association with tumor differentiation rather than tumor size (Supplementary Figure 3).

## Discussion

**FIGURE 4:**
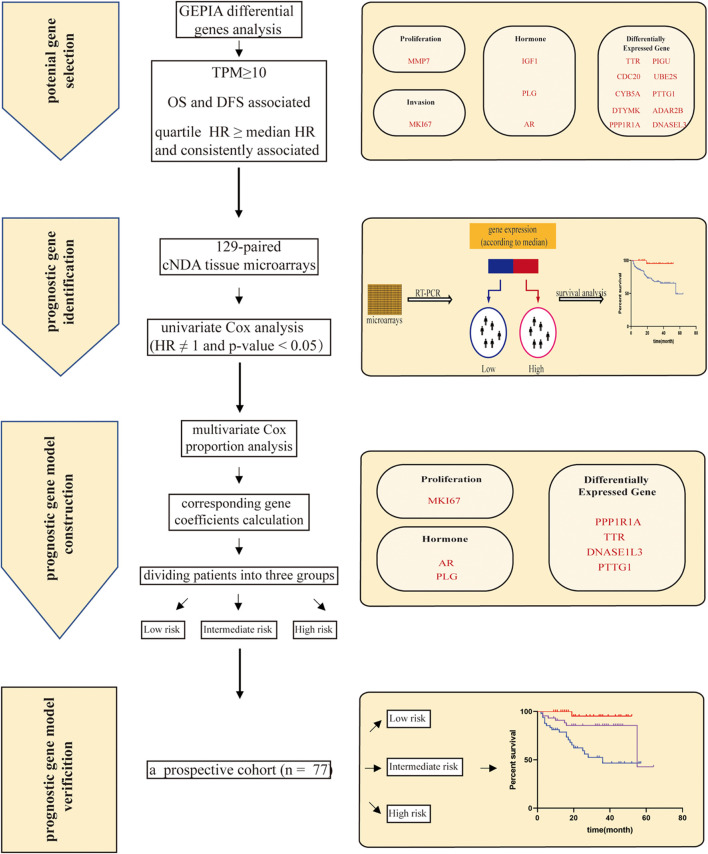
The workflow of our study. First, a list of 15 candidate genes was selected from GEPIA (Gene Expression Profiling Interactive Analysis). Second, a seven-gene signature was identified based on independent survival analysis by Kaplan–Meier curves and a log-rank test. Genes are grouped on the basis of function, covering proliferation: MKI67 (marker of proliferation Ki-67); invasion: MMP7 (matrix metallopeptidase 7); hormone: PLG (plasminogen), IGF1 (insulin like growth factor 1), and AR (androgen receptor); and differentially expressed genes: DNASE1L3 (deoxyribonuclease 1 like 3), PTTG1 (PTTG1 regulator of sister chromatid separation, securin), CDC20 (cell division cycle 20), UBE2S (ubiquitin conjugating enzyme E2 S), PIGU (phosphatidylinositol glycan anchor biosynthesis class U), DTYMK (deoxythymidylate kinase), CYB5A (cytochrome b5 type A), PPP1R1A (protein phosphatase 1 regulatory inhibitor subunit 1A), ADAR2B (adenosine deaminase RNA specific 2b), and TTR (transthyretin). Then, the multivariate Cox proportion model was given to perform the coefficient of the individual gene and calculate a risk score. Risk score = (0.368 × ExprMKI67 + 1.082) + (−0.719 × ExprPTTG1 + 0.424) + (0.286 × ExprDNASE1L3 - 1.276) + (0.204 × ExprPLG + 0.172) + (0.173 × ExprAR - 0.702) + (0.153 × ExprPPP1R1A - 0.731) + (0.286 × ExprTTR - 0.779). The risk score ranged from 1 to 14. Patients were classified into three groups (low, intermediate, and high) by risk score. Finally, the prognostic prediction model of risk scores was verified using an independent prospective cohort.

Due to the high recurrence rate of HCC after surgical resection, proper planning with adjuvant therapies and close monitoring of the patients are routinely required (Ikeda et al., 1993; Tung-Ping Poon et al., 2000; Portolani et al., 2006; Pasini et al., 2020). A precise prognostic model is thus desirable for the patient stratification according to their prognosis to satisfactorily establish a suitable management strategy. It has been consensual that cancer may never completely go away. However, with the appropriate approaches, we can turn cancer into a chronic disease that can be closely managed (Beck and Ng, 2014; Afrasiabi et al., 2020). In this scenario, an evaluation of OS is considered meaningful for the long-term treatment of the cancer patient. For the patients with short OS, postoperative therapies such as transarterial chemoembolization, radiotherapy, and targeted therapy may be beneficial for meaningfully improving the prognosis (Covey and Hussain, 2017; Wang et al., 2017; Huang et al., 2020). In this study, we established a model including seven genes to calculate the risk score and predict the prognosis for the patients with HCC (Figure 4). Our risk score particularly classified patients into a low-, intermediate-, and high-risk group, which strongly displayed correlation with the OS of the patients. The high-risk group typically implied poor survival. Additionally, a separate cohort was carefully constructed to verify the likely value of our predictive signature.

Previous studies promised multiple-gene-expression signatures as the potential model to predict the OS of the patients with HCC and for guiding patient management (Hou et al., 2018; Long et al., 2019; Yan et al., 2019; Zhang et al., 2020; Zhou et al., 2020; Zhu et al., 2020). Individual gene expression signatures in the multiple-gene-expression panel indicated specific behavior to trait tumor evolution. To appropriately predict the OS of the patients with HCC, the predictive systems should replenish certain clinicopathological criteria (Du and Cao, 2012; Quetglas et al., 2014), whereas most of the traditionally existing multiple-gene-prediction signatures were constructed by bioinformatic analysis through public databases or focused on only a specific signal pathway or certain biological processed (Dvorchik et al., 2007; Guan et al., 2019; Dessie et al., 2021). Thus, these existing predictive models are found to be inaccurate or arbitrary sometimes. In addition, some investigations involved too many genes even from different platforms and experiments in signature, which increased the operational cost and was not practical in the clinical settings (Kim et al., 2012; Ouyang et al., 2020). Although the nomogram combining the prognostic gene signature and conventional clinical prognostic factors may have better predictive efficacy than a single biomarker, it only could exhibit a better performance in predicting a short-term survival (1 year and 3 years) but not a long-term survival (5 years) for the patients with HCC (Liu et al., 2019).

The seven specific genes included in our model involved different aspects of tumor progression and seem balanced for adequately reflecting tumor biological features. As an essential gene associated with cell proliferation, *MKI67* expressed in nearly all phases of the cell cycle (Miller et al., 2018). Moreover, *MKI67* has been suggested as an independent survival indicator in the patients with HCC and HCC mouse models (King et al., 1998; Yang et al., 2017, 2018). Similarly, *AR* is a ligand-activated transcription factor, which plays a crucial role in normal liver function and the progression of the liver diseases including HCC (Gelmann, 2002; Ma et al., 2014). High expression of AR in cells, especially in the nuclei, was related to a survival in the patients with HCC (Zhang et al., 2018). Similarly, *PLG* encodes a serine protease called plasminogen that circulates in blood plasma as an inactive zymogen and is converted to plasmin by activators. Moreover, plasminogen located on the cell surfaces is essential for the degradation of extracellular matrices, cell migration, inflammation, oncogenesis, and metastasis, whereas plasmin cleavage releases the angiostatin protein that inhibits angiogenesis (Ponting et al., 1992; Law et al., 2013). A 65-gene-based risk score classifier identified PLG as a predictor of OS in patients with HCC (Kim et al., 2012). The product of *DNASE1L3* (deoxyribonuclease 1-like 3) is a member of the deoxyribonuclease I family that digests DNA in chromatin. This enzyme is required for cytokine secretion following inflammasome activation and plays a role in nuclear endosomal DNA fragmentation during cell apoptosis and necrosis. Its deficiency can lead to anti-DNA responses and autoimmune diseases in humans and mice (Napirei et al., 2005; Al-Mayouf et al., 2011; Sisirak et al., 2016; Shi et al., 2017). However, whether *DNASE1L3* can be a prognostic indicator of HCC is controversial. A study using patients from the TCGA dataset demonstrated that the high expression levels of *STC2*, *CA12*, *CDC20*, *DNASE1L3*, *GBA3*, and *MT1G* in patients with HCC had a significantly shorter survival time (Guan et al., 2019). Nonetheless, a four-novel-gene-based prognostic model to predict the patients with a high-risk HCC excluded *DNASE1L3* because no statistical association was found between *DNASE1L3* and the patient survival (Dessie et al., 2021), while our study sufficiently showed that *DNASE1L3* is an independent prognostic predictor of HCC. *PTTG1* has been found overexpressed in many types of cancer cells, including the hepatoma cell line HepG2 (Fujii et al., 2006). It has been demonstrated to be a multi-faceted gene function in gene regulation, angiogenesis, mitogenesis, cell cycle regulation, chromosomal stability, DNA repair, and apoptosis (Liang et al., 2011). Earlier, *PTTG1* has also been found to be closely connected with angiogenesis, metastasis, and poor prognosis of HCC (Fujii et al., 2006; Tao et al., 2020). *PPP1R1A* encodes protein phosphatase 1 regulatory inhibitor subunit 1A, which is a potent inhibitor of protein phosphatase 1 and regulates hormone metabolism (Luo et al., 2018). By integrated bioinformatics approach, Zhang et al. identified *PP1R1A* as a potential hub gene associated with the pathogenesis and prognosis of HCC (Meng et al., 2020). Likewise, the product of *TTR* (transthyretin) is a serum protein and is synthesized mainly in the liver and is responsible for the transport of thyroxin and retinol binding protein complex to various parts of the whole body. *TTR* also participates in many other biological functions that are directly or indirectly dedicated to tumor progression (Gu et al., 1991; Sharma et al., 2019). It is believed as a novel biomarker for the diagnosis of HCC in cirrhotic patients (Ibrahim et al., 2020). Even though all the above-mentioned genes were individually independent indicators of survival in HCC patients, the joint multi-gene signature should be more convincing and deciphered for the intricate heterogeneity in the patients with HCC; it could be a promising indicator for a long-term survival and guidance for the early treatment.

In summary, here we identified a seven-gene prognostic expression signature by cDNA tissue microarrays for HCC survival analysis. Both model construction and validation were carried out using our clinical microarray data followed by strict RNA quality definition and follow-up endpoint. Consequently, the seven-gene signature presented excellent prediction power in not only a short-term survival but also a long-term overall survival. At the same time, our risk score showed particular correlation with the tumor differentiation stage in patients, quite similar to the real clinical situation. Although the other clinical factors including the presence of existing liver disease, HCC staging, and other postoperative treatment were not excluded, results showed that our seven-gene signature risk model could reflect the present status of patients and performed meaningfully. In fact, the proportion of patients received other therapy after the surgery were balanced in the three groups (about 10%). More importantly, our microarray selection was sternly based on paired tumor and normal tissue samples obtained from the same patients, which was not the case in almost other researches. In addition, compared to the unaffordable costs of using a large gene panel, our approach is convenient, cost-effective, and easy-to-use for clinical practice even in hospitals with limited resources.

However, there are some limitations in our prediction model. First, our cohorts contain only Asian patients, whether our seven-gene signature can be applied to other populations is unknown. Second, gene expression values may be overestimated for a small population in our construction cohort. In our validation cohort, short follow-up time may shrink the difference between the low- and intermediate-risk group. Third, the signature detected prognostic expression in mRNA level; given a divergent gap between mRNA and protein, proteins should be considered in future research as proteins perform the main and ultimate function in biological characteristics. Last but not least, traditional clinical parameters represent the extrinsic manifestation of HCC, whereas gene signatures serve as the intrinsic regulation mechanism of different responses for therapy. Therefore, further study is needed to integrate routine clinical practice with functional gene signature to increase the prognostic potential and guide therapeutic decisions.

## Data Availability Statement

The original contributions presented in the study are included in the article/Supplementary Material, further inquiries can be directed to the corresponding authors.

## Ethics Statement

The studies involving human participants were reviewed and approved by the First Affiliated Hospital, Zhejiang University School of Medicine. The patients/participants provided their written informed consent to participate in this study.

## Author Contributions

TL and QZ conceived the project. QZ and XB designed the experiments. JuW, QZ, FS, JiW, DY, and ZH performed most of the experiments under the supervision of TL. XB, JuW, and QZ performed the bioinformatics analysis. JuW and QZ wrote the manuscript and the other authors made critical revisions. All authors contributed to the article and approved the submitted version.

## Conflict of Interest

The authors declare that the research was conducted in the absence of any commercial or financial relationships that could be construed as a potential conflict of interest.

## Publisher’s Note

All claims expressed in this article are solely those of the authors and do not necessarily represent those of their affiliated organizations, or those of the publisher, the editors and the reviewers. Any product that may be evaluated in this article, or claim that may be made by its manufacturer, is not guaranteed or endorsed by the publisher.

## Supplementary Material

Supplementary Figure 1 A 7-gene signature defined risk scoring was used to demonstrate the disease-free survival of patients with HCC after radical resection. Patients were classified into a low, intermediate, and high-risk group according to the risk scores. **(A)** A retrospective cohort of 129 patients. **(B)** A prospective cohort of 77 patients.

Supplementary Figure 2 A univariate Cox hazard model for the association of clinicopathological characteristics and overall survival in patients with HCC after radical resection. **(A)** A retrospective cohort of 129 patients for tumor size. **(B)** A prospective cohort of 77 patients for tumor size. **(C)** A retrospective cohort of 129 patients for histopathological differentiation. **(D)** A prospective cohort of 77 patients for histopathological differentiation.

Supplementary Figure 3 The correlation between clinicopathological characteristics and risk score in patients with HCC after radical resection. **(A)** A retrospective cohort of 129 patients for tumor size. **(B)** A prospective cohort of 77 patients for tumor size. **(C)** A retrospective cohort of 129 patients for histopathological differentiation. **(D)** A prospective cohort of 77 patients for histopathological differentiation.
