# Supplementary figures and images for: A Seven-Gene Signature to Predict Prognosis of Patients With Hepatocellular Carcinoma
Source: Front Genet. 2021 Sep 16;12:728476. doi: 10.3389/fgene.2021.728476 (PMC8481951; doi:10.3389/fgene.2021.728476)

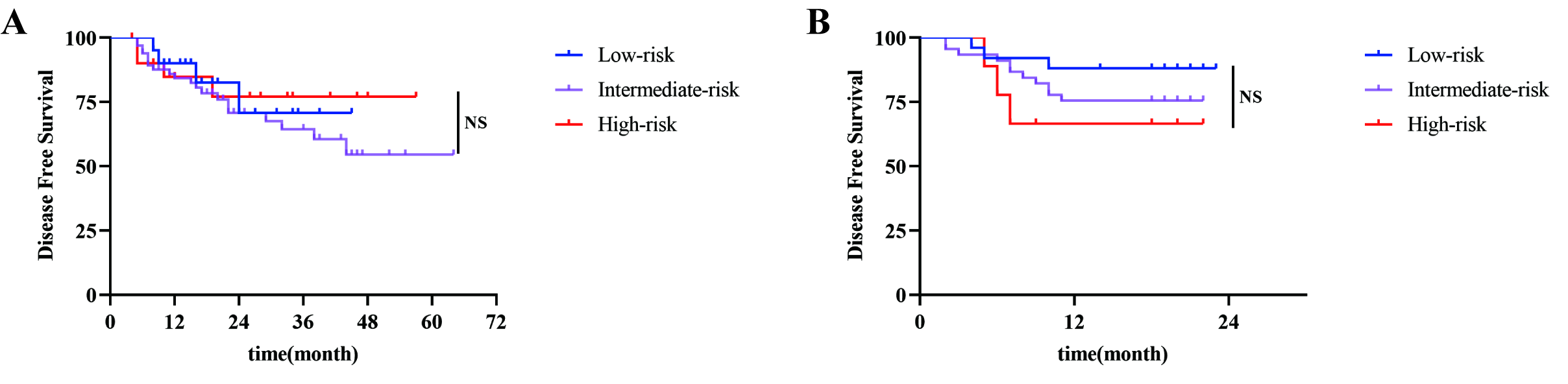

Supplement: Supplementary Figure 1 — A 7-gene signature defined risk scoring was used to demonstrate the disease-free survival of patients with HCC after radical resection. Patients were classified into a low, intermediate, and high-risk group according to the risk scores. (A) A retrospective cohort of 129 patients. (B) A prospective cohort of 77 patients. [file Image_1.tif]

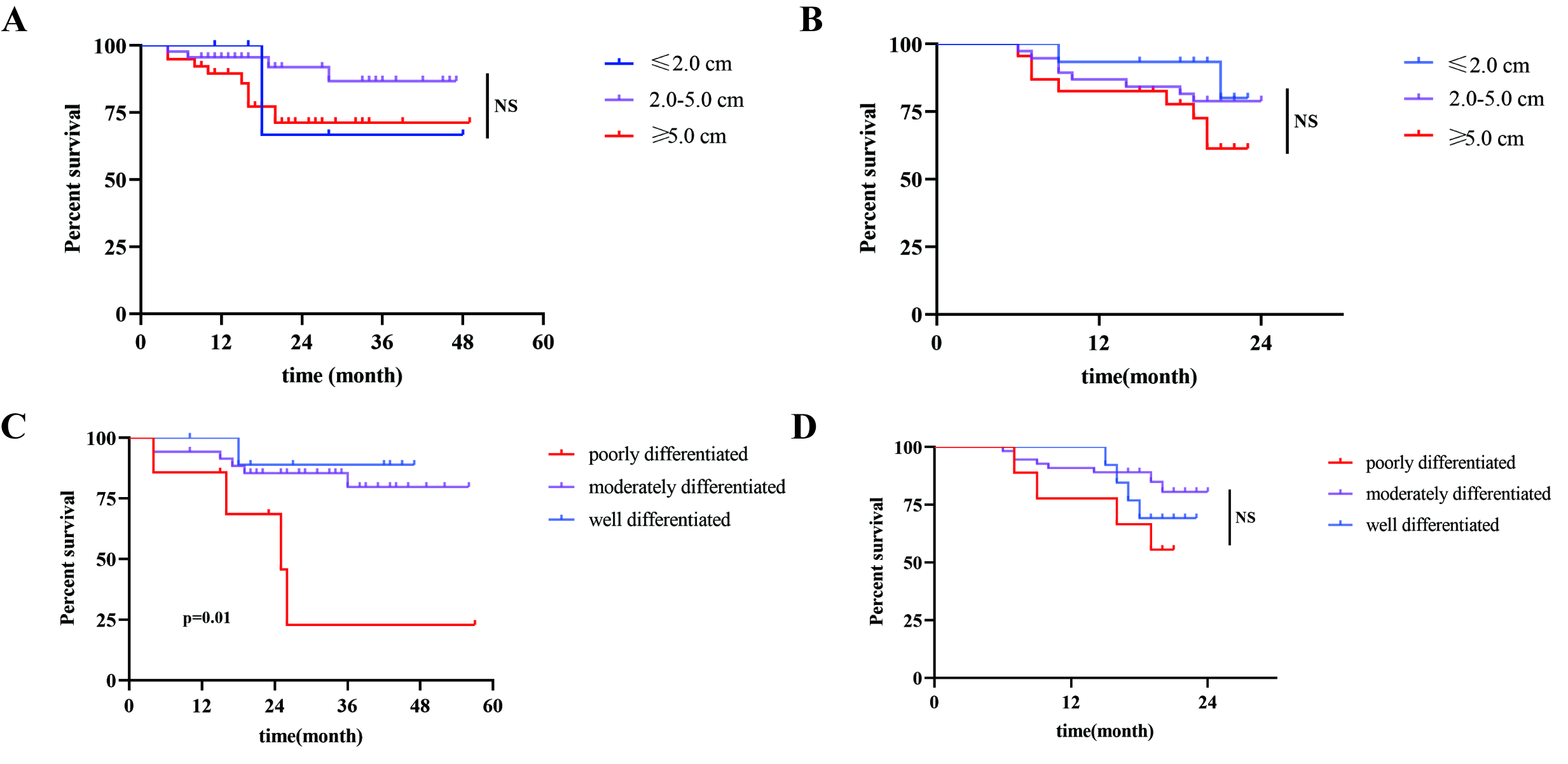

Supplement: Supplementary Figure 2 — A univariate Cox hazard model for the association of clinicopathological characteristics and overall survival in patients with HCC after radical resection. (A) A retrospective cohort of 129 patients for tumor size. (B) A prospective cohort of 77 patients for tumor size. (C) A retrospective cohort of 129 patients for histopathological differentiation. (D) A prospective cohort of 77 patients for histopathological differentiation. [file Image_2.tif]

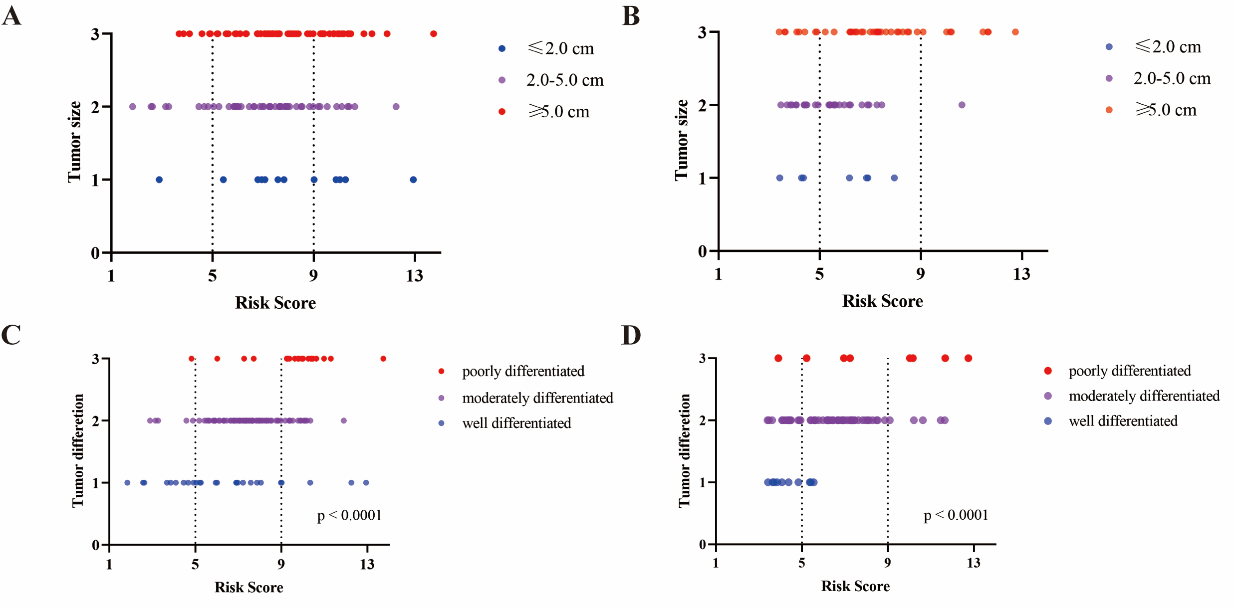

Supplement: Supplementary Figure 3 — The correlation between clinicopathological characteristics and risk score in patients with HCC after radical resection. (A) A retrospective cohort of 129 patients for tumor size. (B) A prospective cohort of 77 patients for tumor size. (C) A retrospective cohort of 129 patients for histopathological differentiation. (D) A prospective cohort of 77 patients for histopathological differentiation. [file Image_3.tif]

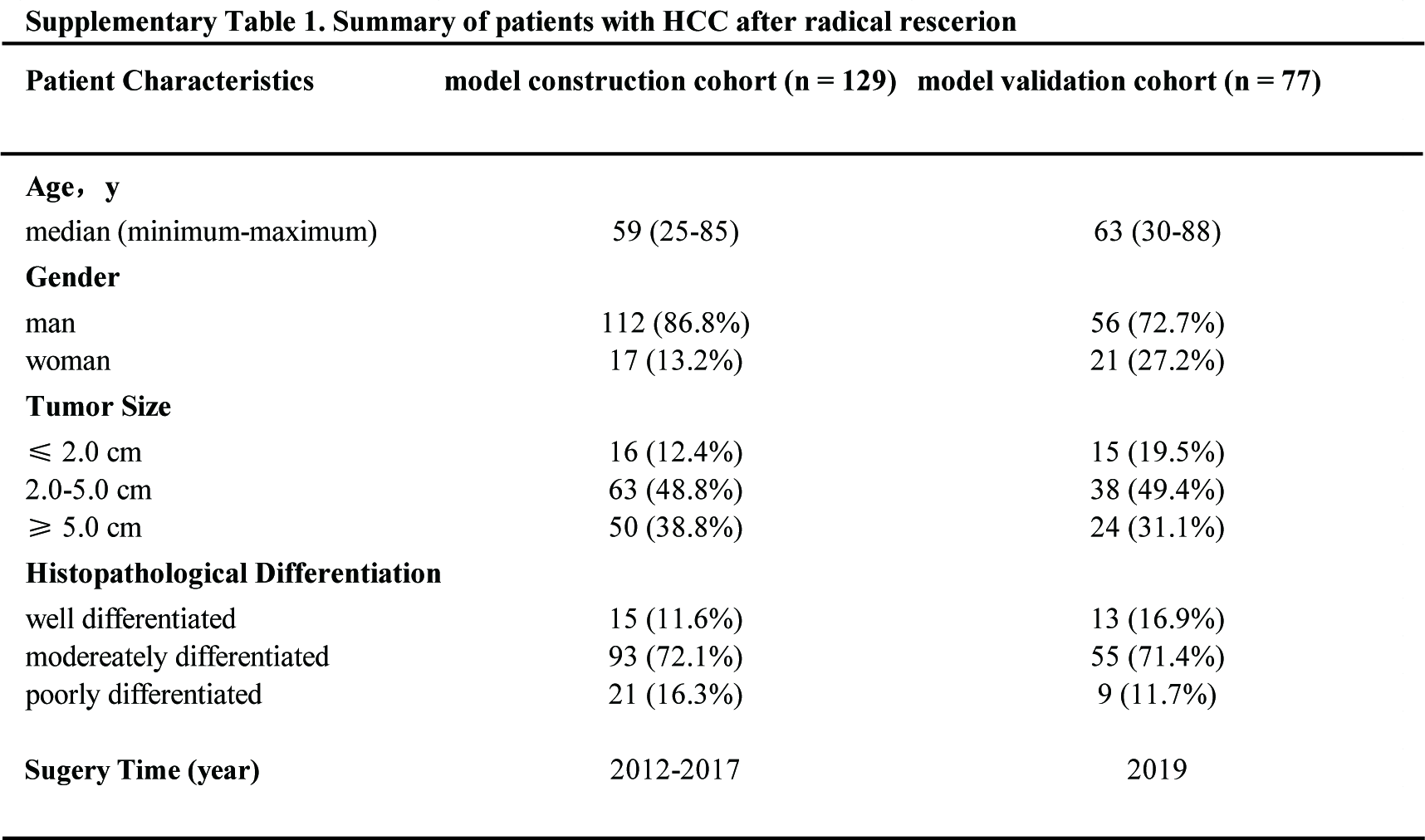

Supplement: Supplementary file 4 [file Data_Sheet_1.zip › Suppl Table 1.TIF]

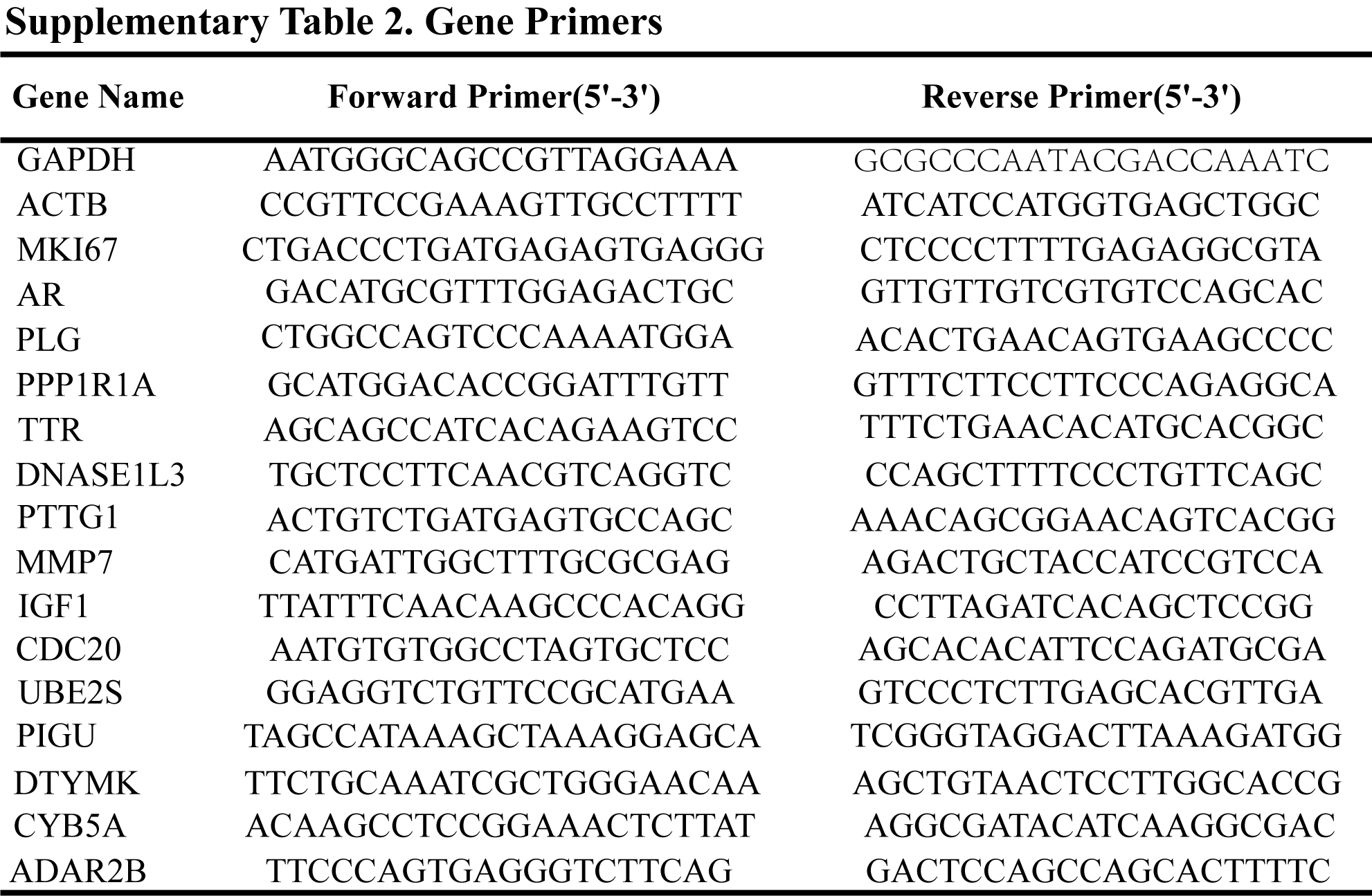

Supplement: Supplementary file 4 [file Data_Sheet_1.zip › Suppl Table 2.TIF]
